# Patient anxiety of verticalization on day 0 after a Cesarean section

**DOI:** 10.1007/s00404-020-05748-3

**Published:** 2020-08-27

**Authors:** Anna Prokopowicz, Aleksandra Korzeniewska, Katarzyna Byrka

**Affiliations:** 1 Division of Practical Obstetrics, Department of Gynaecology and Obstetrics, Faculty of Health Sciences, Wroclaw Medical University, ul. Bartla 5, 51-618 Wrocław, Poland; 2 Department of Family Medicine, Wroclaw Medical University, Wrocław, Poland; 3 Faculty of Psychology, SWPS University of Social Sciences and Humanities, Wrocław, Poland

**Keywords:** Anxiety, Cesarean section, Mobilization, Verticalization, Post-operative period

## Abstract

**Purpose:**

The ERAS protocol recommends fast mobilization of a patient along with providing psychological comfort; however, early verticalization can generate mental discomfort. In the post-operative period after Caesarean section (CC), a patient may experience pain, anxiety and negative affect. The main aim of the study was to investigate levels of patient anxiety concerning verticalization on day 0 after CC using. Also, the dependence between anxiety of verticalization and the following: pain, anxiety as a permanent disposition, resistance to pain, negative and positive emotions, and positive orientation was described.

**Methods:**

The study included 150 women on day 0 after their CC. The measurement of anxiety concerning verticalization, pain, and also negative and positive emotions was conducted twice. During the first measurement, variables such as anxiety levels as a constant disposition, level of pain resistance and positive orientation were also monitored.

**Results:**

Patients upon arrival at the post-operative room declared a higher level of anxiety of verticalization (*p* < 0.05) (Median = 4; IQR = 5) when compared to 6 h after surgery (Median = 3; IQR = 4). A weak correlation of anxiety and pain was noted (*r* = 0.264; *p* < 0.01) in the second measurement. In both measurements, negative emotions and pain resistance proved to be the strongest variables explaining anxiety concerning verticalization.

**Conclusions:**

Low levels of pain experienced by a patient after CC do not explain the variance in anxiety of verticalization. The use of the Numerical Rating Scale (NRS) of anxiety allows care givers to gain patients' psychological perspective in different moments after CC.

## Introduction

A Cesarean section is currently the most frequently performed obstetric surgery, and every fifth newborn is born with the use of a scalpel [1]. Most Cesarean section patients receive a conduction anesthesia, which is a type of anesthesia that removes all pain in the operating area but leaves patients aware. Its total analgesic effect lasts for several hours. After it fades away, patients begin to experience severe post-operative pain, which is then alleviated by multimodal analgesia [2]. According to the standards of the enhanced recovery after surgery (ERAS) protocol, multimodal analgesic therapy allows for maximum reduction of pain. At the same time, patients stay aware, which increases the chances of early verticalization [3, 4]. The main purpose of rapid post-operative verticalization is the prophylaxis of thrombophlebitis, prevention of respiratory and digestive complications, prevention of post-operative adhesions and the improvement of blood supply to tissues [5]. The study of Borges et al. and their review of current research concerning post-operative pain after a Cesarean section clearly showed that regardless of the analgesic therapy used, most patients after a Cesarean section suffer from severe pain [6].

Research has shown that one of the predictors of the level of pain experienced in the post-operative period after different surgeries is perioperative anxiety [6–9]. We distinguish two types of anxiety. The first is a basic and stable level of anxiety, a so-called trait anxiety that originates from genetics and is shaped by specific life experiences. The second type is a transient level of anxiety, a state anxiety that is felt depending on exposure to various acute aversive situations. A state anxiety is characterized by high variability depending on the subjective perception of various types of threatening factors, which in turn may depend on the level of individual trait anxiety [10, 11].

Pinto et al., reviewed research concerning the effects of preoperative anxiety on pain levels and the process of patient improvement after orthopaedic surgery [9]. A higher level of experienced anxiety was correlated with a higher level of post-operative pain and with a less effective recovery process. Importantly, anxiety can be generated and enhanced by mere awareness of the inevitability of pain, especially when patients engage in catastrophic thinking and assess themselves as having poor pain resistance [12–14].

To our knowledge, so far, anxiety and experienced pain have not been examined in the post-operative period. Understanding the role of anxiety and pain is particularly important in the context of patient recovery after a Cesarean section, because early verticalization may be beneficial for themselves and for newborns, as their mothers may take care of their infants more quickly. The aim of this research was to test the relationship between anxiety of verticalization and pain in patients after a Cesarean section in the first 6 h after surgery using two different points of measurement. In addition, we examined the effect of anxiety as a trait, and negative affect on the acute anxiety of verticalization. In opposition to anxiety, positive affect and positive orientation are considered to be predictors of health and a faster recovery after illness [15–17]. We thus expected that pain resistance and positive affect would mitigate the acute anxiety generated by expected verticalization. Additionally, we used adjustments for variables such as the order of Cesarean sections that might also explain experienced anxiety.

## Methods

### Population and settings

The research was conducted in the Postoperative Room at the University Clinical Hospital in Wroclaw. The study included 150 women on day 0 after Cesarean surgery from 15.12.2017 to 27.05.2018. During the examination after the surgery, the operating team recommended each patient a verticalization 6 h after anesthesia (after about 5 h of staying in the recovery room). All the examined women had conduction anesthesia during their Cesarean section. Then, all of them underwent the same analgesic therapy.

Immediately after arriving at the post-operative room, the women became acquainted with the examination procedure and signed a written informed consent to participate in the research. The study was approved by the ethics committee of the SWPS University of Social Sciences and Humanities in Wrocław (Decision 05P/12-2017). The data analyzed in this paper is part of the larger research project described in an unpublished diploma thesis. All the surveys and questionnaires that were used in the study were completed anonymously.

Clinical data of patients were summarized using descriptive statistics. Obstetric data were summarized using the Robson classification [18, 19]. The following observed variables were operationalized using the self-description method: anxiety as a trait, anxiety as a state, pain, negative affect, subjective pain resistance, and positive orientation. Anxiety, pain, and also positive and negative affect were measured twice at the same time: upon arrival at the recovery room (T1) and 5 h after arrival (about 6 h after anesthesia, T2). Patients between the T1 measurement (after completing the questionnaires) and the T2 measurement were given their children for kangaroo mother care (kangaroo mother care—early skin-to-skin contact between a mother and her newborn). The following individual variables as permanent dispositions: trait anxiety, subjective pain resistance, and positive orientation were measured only once—immediately after arrival in the post-operative room.

### Questionnaires

The level of state anxiety and pain were measured using the Numerical Rating Scale (NRS) from 0 to 10. Studies show that the validity and reliability of these scales enable them to be used for scientific research [11, 20].

When assessing pain using the NRS, 0 means a complete absence of pain, and 10 corresponds to the most severe pain imaginable. For the purpose of this study, when considering anxiety in the NRS, 0 means a complete absence of anxiety of verticalization, and 10 refers to the strongest anxiety of verticalization imaginable.

Additionally, the level of anxiety as a permanent disposition was measured in patients using the Polish adaptation of Spielberger’s State-Trait Anxiety Inventory (STAI) [21]. Participants responded on a 4-point scale with options ranging from 1 to 4, where 1 means ‘almost never’ and 4 ‘almost always’. The questionnaire consists of 13 positively formulated items and 7 negatively formulated items.

The intensity of negative and positive affect was measured using the International Positive and Negative Affect Schedule Short-Form (PANAS S-F) [22]. Five negative affective states, such as feeling afraid, ashamed, hostile, nervous and upset, and five positive ones, such as being active, alert, attentive, determined and inspired, were analyzed. Respondents were asked to indicate the intensity of these conditions on a five-point scale ranging from ‘very weak’ (1) to ‘very strong’ (5).

Positive orientation was measured using eight statements from a Polish adaptation of a questionnaire, which examined the functioning of a person in certain situations with regards to belief in their own resources using a five-point scale ranging from ‘definitely disagree’ (1) to ‘definitely agree’ (5) [23].

Data concerning subjective pain resistance were also collected using the authors’ self-made questionnaire, in which patients were asked to mark the correct completion of their opinion about themselves: I believe that I am resistant to pain on a five-point scale ranging from ‘Much less than an average patient’ (1) to ‘Much more than an average patient’ (5).

The reliability indicator for the measures used in the study was examined. The reliability statistics (Cronbach’s α) for the scales were as follows: anxiety measured STAI-trait α = 0.85, affect measured I-PANAS: positive affect in T1 measurement α = 0.76, in T2 measurement α = 0.78, negative affect in T1 measurement α = 0.72, in T2 measurement α = 0.70, positive orientation α = 0.65.

IBM SPSS Statistics 25 software (IBM Corp., Armonk, NY, USA) was used for the statistical analysis. The distribution of variables was examined using the Kolmogorov–Smirnov test. The obtained statistical values showed that all variables, except positive affect in the T1 measurement, presented significant differences from the normal distribution. For intergroup comparisons, the non-parametric Mann–Whitney *U* test was used, and for comparisons of dependent measurements, the Wilcoxon signed-rank test was used. For correlation analysis, Spearman’s rank correlation coefficient was used. To extract predictors of anxiety of verticalization, multivariate regression analyses of anxiety were performed in both measurements. Finally, a post-hoc power analysis was conducted [24].

## Results

### Population

The study involved 150 women with an average age of 32 ± 4.6 years (range: 20–48). Among them, 126 patients (84%) declared higher education, 19 patients (12.7%) declared secondary education, 2 patients (1.3%) declared vocational education, 1 patient (0.7%) declared junior high education, and 2 patients (1.3%) declared primary education. In the examined group, 127 women underwent Cesarean sections in elective mode (84.7%), and 23 (15.3%) in urgent mode. In 71 patients, the Cesarean procedure was performed for the first time (47.3%), and for 79 women (52.7%) as a subsequent time. Overall, 127 (84.7%) women provided kangaroo mother care for their children, and 23 patients (15.3%) did not provide such care due to their child’s state of health.

The studied population of patients was also characterized using the widely used Robson classification—the Ten Group Classification System (TGCS). The highest number of patients (50.5%) were classified to group 5 (all multiparous with at least one previous uterine scar, with single cephalic pregnancy, ≥ 37 weeks gestation). Next, with a decreasing order were the following groups: group 2 with 17.3% of women (nulliparous with single cephalic pregnancy, ≥ 37 weeks gestation who either had labour induced or delivered by Cesarean section before labour), group 6 with 16% of women (all nulliparous women with a single breech pregnancy), group 11 with 7.3% of women (all women with a single cephalic pregnancy, < 37 weeks gestation, including women with previous scars, group 4 with 4.7% of women (multiparous without a previous uterine scar, with single cephalic pregnancy, ≥ 37 weeks gestation who either had labor induced or delivered by Cesarean section before labor), group 1 with 2% of women (nulliparous with single cephalic pregnancy, ≥ 37 weeks gestation in spontaneous labor), group 7 with 1.3% of women (all multiparous women with a single breech pregnancy including women with previous uterine scars) and finally group 3 with 0.7% of women (multiparous without a previous uterine scar, with single cephalic pregnancy, ≥ 37 weeks gestation in spontaneous labor).

### Comparison of anxiety of verticalization and pain in two measurements

The anxiety of verticalization and pain differed in both measurements. Anxiety decreased 6 h after surgery when compared to the measurement upon arrival at the observation room [Z (149 = 2.689; *p* = 0.007; (*p* < 0.01)], despite the increase in pain [Z (149) = 7.903; *p* = 0.000; (*p* < 0.01)].

### Anxiety of verticalization and other variables

Descriptive statistics of anxiety, pain, affect, pain resistance, and positive orientation are presented in Table 1. Both measurements of anxiety were highly correlated with each other. Anxiety in the first measurement was highly correlated with negative affect. The strength of other relations was mainly low or average. Anxiety of verticalization only correlated with pain 6 h after surgery. Correlations of anxiety variables is presented in Table 2.

**Table 1:** Descriptive statistics of anxiety, pain, affect, pain resistance, and positive orientation (*n* = 150)

	*M*	Median	SD	IQR	*D*
Anxiety of verticalization upon arrival at the recovery room	3.99	4.0	3.02	5	0.15**
Anxiety of verticalization in the first 6 h after surgery	3.54	3.0	2.88	4	0.14**
Trait anxiety	36.17	36.0	6.92	9	0.08**
Pain upon arrival at the recovery room	0.57	0.00	1.43	1	0.40**
Pain in the first 6 h after surgery	2.25	2.0	1.67	2	0.15*
Pain resistance	3.05	3.0	0.83	1	0.28**
Positive orientation	33.32	33.0	3.38	5	0.09**
Positive panas upon arrival at the recovery room	16.10	16.0	3.37	5	0.07
Negative panas upon arrival at the recovery room	10.73	11.0	3.41	5	0.90**
Positive panas in the first 6 h after surgery	15.52	15.50	3.55	5	0.08*
Negative panas in the first 6 h after surgery	9.04	9.00	3.04	4	0.12**

* *p* < 0.05; ** *p* < 0.01

**Table 2:** Correlations with post-hoc power calculations between anxiety of verticalization upon arrival at the recovery room (T1) and in the first 6 h after surgery (T2), and trait anxiety, pain, pain resistance, affect, and positive orientation

Spearman’s rho	Anxiety T1	Anxiety T2	Power T1	Power T2
Anxiety of verticalization upon arrival at the recovery room	–	0.754**	–	1
Anxiety of verticalization in the first 6 h after surgery	0.754**	–	1	–
Trait anxiety	0.290**	0.227**	0.953	0.802
Pain po upon arrival at the recovery room	0.146	0.112	0.432	0.277
Pain in the first 6 h after surgery	0.076	0.264**	0.152	0.908
Pain resistance	− 0.344**	− 0.281**	0.992	0.94
Positive orientation	− 0.277**	− 0.199*	0.933	0.689
Positive affect upon arrival at the recovery room	− 0.168*	− 0.151	0.541	0.456
Negative affect upon arrival at the recovery room	0.502**	0.353**	1	0.994
Positive affect in the first 6 h after surgery	− 0.024	− 0.081	0.06	0.167
Negative affect in the first 6 h after surgery	0.357**	0.421**	0.995	1

* *p* < 0.05; **p < 0.01

In both measurements, it turned out that the higher the level of negative affect and trait anxiety, the higher the anxiety of verticalization. However, in both measurements, anxiety correlated with pain resistance and with a positive orientation—the more the patient imagined that she was less resistant to pain than the average patient and had less faith in her own capabilities, the stronger the level of anxiety she felt concerning verticalization (Table 2).

### Intensification of anxiety of verticalization versus the order of Cesarean sections

Women who underwent more than one Cesarean surgery felt greater anxiety of verticalization 6 h after their Cesarean section (Mdn = 4.00; Mrang = 83.75) than women who had their first Cesarean section (Mdn = 3.00; Mrang = 66.32) (Table 3).

**Table 3:** Anxiety of verticalization and order of Cesarean sections

	First section (*n* = 71)		Subsequent section (*n* = 79)		*U*	*p*
	Mdn	Mrang	Mdn	Mrang		
Anxiety of verticalization upon arrival at the recovery room	3.00	70.38	3.00	80.10	2441.00	0.168
Anxiety of verticalization in the first 6 h after surgery	3.00	66.32	4.00	83.75	2153.00	0.013*

* *p* < 0.05; **p < 0.01

### Predictors of anxiety of verticalization

The next stage of the analysis was an attempt to isolate predictors of anxiety of verticalization after arriving at the post-operative room and also 6 h after surgery. For this purpose, two multivariate regression analyses were performed separately for each anxiety measurement.

In the first measurement, the group of explanatory variables included the following indicators: trait anxiety (*β* = 0.17; *p* = 0.073), pain resistance (*β* = − 0.22; *p* = 0.004), negative affect (*β* = 0.38; *p* = 0.000), positive affect (*β* = − 0.07; *p* = 0.32), and positive orientation (*β* = 0.02; *p* = 0.849) (Table 4). The model explains 33% of the variances of anxiety of verticalization.

**Table 4:** Regression model along with effect size and power calculation—predictors of anxiety of verticalization upon arrival at the recovery room (T1)

Predictors	B (SE)	β	*p* value
Trait anxiety	0.076 (0.04)	0.17	0.073
Pain resistance	− 0.785 (0.27)	− 0.22	0.004**
Negative affect	0.342 (0.06)	0.38	0.000**
Positive affect	− 0.064 (0.07)	− 0.07	0.32
Positive orientation	0.02 (0.09)	0.02	0.849
*F*	14.137		0.000**
*R*^2^	0.33		
*f*^2^	0.49		
Power	1		

* *p* < 0.05; **p < 0.01

In the second measurement, the group of explanatory variables included the following indicators: pain (*β* = 0.15; *p* = 0.043), trait anxiety (*β* = 0.17; *p* = 0.077), pain resistance (*β* = − 0.19; *p* = 0.009), negative affect (*β* = 0.31; *p* = 0.000), and also positive orientation (*β* = 0.02; *p* = 0.875) (Table 5). The model explains 29% of the variances of anxiety of verticalization.

**Table 5:** Regression model along with effect size and power calculation—predictors of anxiety of verticalization in the first 6 h after surgery (T2)

Predictors	B (SE)	*β*	*p* value
Trait anxiety	0.074 (0.04)	0.17	0.077
Pain	0.264(0.13)	0.15	0.043*
Pain resistance	− 0.673 (0.26)	− 0.19	0.009**
Negative affect	0.292 (0.07)	0.31	0.000**
Positive orientation	0.013 (0.85)	0.02	0.875
*F*	11.575		0.000**
*R*^2^	0.287		
*f*^2^	0.402		
Power	0.999		

* *p* < 0.05; **p < 0.01

Both models show that the stronger the negative affect the patient felt and the more she thought that she was not resistant to pain, the stronger the observed anxiety of verticalization. At a pain level of *M* = 2.25 6 h after surgery, pain in this model was an explanatory variable on the border of significance (*β* = 0.15; *p* = 0.043).

### Pain resistance versus affect, positive orientation and anxiety as a permanent disposition

Spearman’s rho correlation analysis was conducted for pain resistance with trait anxiety, affect, and positive orientation. Negative correlations were found with trait anxiety (rho = − 0.226; *p* < 0.01), and negative affect (rho = − 0.300; *p* < 0.01) and positive correlations were indicated with positive orientation (rho = 0.285; *p* < 0.01). The strength of these correlations was weak.

### Post-hoc power analysis

Results of power calculations for correlations are included in Table 2. Only two significant correlations had power below 0.8, even though they were statistically significant; the first between positive orientation and anxiety T2, the second between positive affect upon arrival at the recovery and anxiety T1. Other significant correlations have post-hoc power above 0.8. Assuming effect size d, which equals 0.3 (small effect size) and the number of participants from our sample (150 participants), power for Wilcoxon signed-rank test is 0.94. Therefore, the reported sample appears to be enough for conclusions based on the Wilcoxon signed-rank test. Finally, we applied two post-hoc power calculations (see Tables 4 and 5) for two regression models predicting the anxiety of verticalization in two-time stamps based on the observed f2 effect sizes. Both of those models had power near 1.

## Discussion

The objective of this research was to test to what extent patients feel anxiety after a Cesarean section with regard to the recommended early initial verticalization, what affects the level of expected anxiety, and whether the anxiety of verticalization changes over time. The analyses showed that patients experience anxiety of verticalization in both measurements at extremely low levels of pain. Anxiety decreased 6 h after surgery (T2), despite the increase in pain in this measurement, when compared to the anxiety after arriving at the post-operative room (T1), when patients mostly did not feel any pain. In the first measurement (T1), over 40% of patients experienced a higher level of anxiety of verticalization (and did not feel any pain) than the average level of anxiety in their group. Levels of anxiety of verticalization in both measurements positively correlated with each other and showed weak correlations with trait anxiety.

The strongest predictors of anxiety of verticalization were negative affect and the perception of oneself as being less resistant to pain than the average patient. The pain did not explain the level of anxiety in the first measurement, mostly because there was no variance in experienced pain. In the second measurement, it was on the border of significance. In scientific literature, no publications on norms of anxiety of verticalization after a Cesarean section were found, so it is therefore difficult to find a reference point for the levels of anxiety in the examined group. When using studies in which the results of anxiety are cited as a condition of patients after a Cesarean section that is measured using the VAS scale, a large discrepancy of anxiety levels in the post-operative period can be found, which ranges from 1.2 to 5.8 points [25, 26].

In the present research, it was expected that anxiety in the T2 measurement would increase with an increase in pain when compared to the T1 measurement. However, the anxiety diminished despite the increase in pain. This can be explained by the low level of pain in the T2 measurement (*M* = 2.25; Mdn = 2.0) in relation to the post-operative pain given in the study of Borges et al. on a group of 1062 women after Cesarean surgery, which ranged from 6.6 at the worst moment to 3.3 when experiencing the least pain [6]. This fact could explain a change in the levels of anxiety opposite to the hypothesis of the present study. The decrease in anxiety of verticalization can also be explained by the fact that the majority of mothers (*n* = 127; 84.7%) provided kangaroo mother care to their children. Also, other studies show that kangaroo mother care stimulates a decrease in anxiety in mothers [27].

The results of the level of felt pain in the present study show that the examined patients received very effective pain therapy. This fact may explain the change in anxiety levels, which is inconsistent with the hypothesis of the present research. Moreover, our study revealed that perceiving oneself as a person less resistant to pain than other patients explains the variance of anxiety of verticalization. This result is consistent with studies that showed those non-adaptive beliefs about the perception of a given situation as difficult or unbearable due to pain could worsen the process of motor rehabilitation and influence stronger pain sensations [9, 28].

The feeling of negative affect was most strongly associated with the anxiety of verticalization. The research of Cohen et al. shows that a high level of negative affect explains the variance of social anxiety and depression [29]. Other studies show that pre-operative anxiety correlates with post-operative pain and affects post-operative pain after a Cesarean section [6, 30]. In the present study, a positive correlation of anxiety and pain was also obtained in the second measurement. However, the anxiety of verticalization after arriving at the post-operative room (T1) did not correlate with the pain 6 h after the surgery (T2).

In the multi-shift regression model, pain explained the variance of the felt anxiety in the T2 measurement on the border of significance. It is difficult to discuss this result as no studies have been found that evaluate the effect of acute post-operative pain on anxiety in the post-operative period. However, the direction of exploration of the impact of acute pain on anxiety cannot be overlooked. Lumley et al., in a review of research on the interaction of pain and affect, show that persistent pain affects the feeling of anxiety and fear [31]. It is worth noting that about 70% of the variance of anxiety of verticalization has not been explained. The explanation of the missing variance may be an inspiration for further research.

Obtaining psychological support concerning anxiety of verticalization in a hospital seems justified when bearing in mind research that described the destructive role of anxiety in the Chronic Low Back Pain (CLBP) model. The CLBP model assumes that anxiety of pain is associated with avoiding movement that causes pain [28]. Similarly, the anxiety of verticalization after Cesarean section surgery can mean that patients avoid movement and, as a result, transform acute pain into chronic pain. The research of Pinto et al. showed that one of the predictors of the transformation of acute pain into chronic pain in patients after a hysterectomy was perioperative anxiety [9]. In addition, anxiety of movement that causes pain can reduce the motivation of women to perform necessary tasks during the puerperium period. A review of research concerning anxiety of pain generated by movements shows that patients, whose main goal is to avoid pain, give up trying to achieve other important life goals [32].

Many variables may have an impact on the level of pain in patients after Cesarean section. The present study was devoted to psychophysical well-being of patients in the post-operative period of the Cesarean section; hence, it does not apply to the assessment of medical indications and the analysis of Caesarean section features. Nevertheless, it is worth noting that group 5, according to the Robson classification, which included patients following at least one Cesarean section, was the biggest. This result is in line with the trend observed in other clinical research on the reduction of the Cesarean section epidemic [18]. The second method of analyzing the reasons for the increase in the number of Caesarean sections is the evaluation of indications for this type of delivery in women after Caesarean section classified as groups 1 and 3 who had undergone Caesarean section for the first time [19]. The assessment of relationships between variables associated with Cesarean section should be the topic of further research. An extensive evaluation of anxiety before delivery in pregnant women may deliver new data and can help to develop methods of interventional work for people with a high level of anxiety.

Finally, the study of anxiety levels in mothers after giving birth seems an important issue, because anxiety correlates with depression. Separating postpartum anxiety from postpartum depression is difficult and both may have a destructive effect on the whole of the broadly understood period of motherhood [33, 34].

### Limitations

The main limitation of the project was the fact that general state anxiety was not examined with a validated scale in order to check the correlation with the anxiety of verticalization. Research by Pinto et al. shows that state anxiety, measured using the Visual Analogue Scale (VAS) from 0 to 10, highly correlates with state anxiety measured using STAI—the state in women after a hysterectomy [9]. Anxiety of verticalization in the present study, measured using the NRS from 0 to 10, correlated with trait anxiety measured using the STAI-trait. This result shows consistency with the study of Davey et al., in which state anxiety in women awaiting breast surgery, measured using VAS from 0 to 10, correlated with trait anxiety measured using STAI [35].

In addition, the authors use a question about pain resistance without prior validation of this one-item measurement. However, a one-item question on the Likert scale can quickly supplement knowledge concerning the patient's subjective perspective, as is the case when using a long questionnaire [35]. Subjecting a one-item question concerning pain resistance and the NRS of anxiety of verticalization to the validation process may be an inspiration for further research.

Finally, the fact that most of the assessed women had a higher education and a planned Cesarean section, and also the fact that the research was conducted in only one center, warrants caution when generalizing our results to a wider population.

## Conclusions

Our study deepens the understanding of the role of anxiety in the process of verticalization. We found that subjective thinking (conviction) about oneself as a patient with low pain resistance and feeling a higher level of negative affect can cause an increase in the level of anxiety of verticalization. Low levels of pain felt by a patient after a Cesarean section explain, to a small extent, the variance of anxiety of verticalization. Despite the mentioned limitation, the use of simple one-item questions or scales has huge practical potential. By using them, medical staff can quickly supplement knowledge about patients’ subjective psychological perspective and adapt the treatment. It is probable that patients with a high level of anxiety in relation to the reference group require additional attention and support from medical staff.

## Data Availability

My manuscript has no associated data.
